# Transcriptome analysis of the sex pheromone gland of the noctuid moth *Heliothis virescens*

**DOI:** 10.1186/1471-2164-11-29

**Published:** 2010-01-14

**Authors:** Heiko Vogel, Andrew J Heidel, David G Heckel, Astrid T Groot

**Affiliations:** 1 Max Planck Institute for Chemical Ecology, Department of Entomology, Hans Knoell Strasse 8, 07745 Jena, Germany; 2 Leibniz Institute for Age Research, Fritz Lipmann Institute (FLI), Beutenbergstraße 11, 07745 Jena, Germany

## Abstract

**Background:**

The chemical components of sex pheromones have been determined for more than a thousand moth species, but so far only a handful of genes encoding enzymes responsible for the biosynthesis of these compounds have been identified. For understanding the evolution of moth sexual communication, it is essential to know which genes are involved in the production of specific pheromone components and what controls the variation in their relative frequencies in the pheromone blend. We used a transcriptomic approach to characterize the pheromone gland of the Noctuid moth *Heliothis virescens*, an important agricultural pest, in order to obtain substantial general sequence information and to identify a range of candidate genes involved in the pheromone biosynthetic pathway.

**Results:**

To facilitate identifying sets of genes involved in a broad range of processes and to capture rare transcripts, we developed our majority of ESTs from a normalized cDNA library of *Heliothis virescens *pheromone glands (PG). Combining these with a non-normalized library yielded a total of 17,233 ESTs, which assembled into 2,082 contigs and 6,228 singletons. Using BLAST searches of the NR and Swissprot databases we were able to identify a large number of putative unique gene elements (unigenes), which we compared to those derived from previous transcriptomic surveys of the larval stage of *Heliothis virescens*. The distribution of unigenes among GO Biological Process functional groups shows an overall similarity between PG and larval transcriptomes, but with distinct enrichment of specific pathways in the PG. In addition, we identified a large number of candidate genes in the pheromone biosynthetic pathways.

**Conclusion:**

These data constitute one of the first large-scale EST-projects for Noctuidae, a much-needed resource for exploring these pest species. Our analysis shows a surprisingly complex transcriptome and we identified a large number of potential pheromone biosynthetic pathway and immune-related genes that can be applied to population and systematic studies of *Heliothis virescens *and other Noctuidae.

## Background

Moth sexual communication has been a major research focus for understanding the evolution of prezygotic isolation [1-7]. This is because moth sexual communication is primarily chemical, and thus variation at the chemical level provides the basis for evolutionary change. The females produce and emit pheromone from a specialized structure, the sex pheromone gland (PG) at the tip of their abdomen [8-10], while the males perceive and respond to these pheromones from a distance [11]. Since the first identification of the sex pheromone in *Bombyx mori *[12], sex pheromones and attractants of about 1,600 moth species have been chemically identified [13]. Most moth pheromone components are even-numbered C_10_-C_18 _straight-chain, unsaturated derivatives of fatty acids, with the carbonyl carbon modified to form an oxygen-containing functional group (alcohol, aldehyde, or acetate ester) [14,15]. Species-specificity of female pheromone blend production is the result of the combination of two or more compounds and the ratio between them. Males also exhibit species-specificity in the behavioral attraction to this particular blend [2,4].

The biosynthetic pathways of many sex pheromones have been partly elucidated [14,16-18]. Free saturated fatty acids are produced *de novo *and converted to their acyl-CoA thioesters before being incorporated into glycerolipids or converted to pheromone [19]. Despite knowledge of the chemical intermediates, only a few enzymes in the biosynthetic pathways have been identified and characterized. Most emphasis has been on the identification and characterization of desaturases, enzymes that introduce a double bond at a specific position in the carbon chain. So far Δ9, Δ10, Δ11, Δ12 and Δ14 desaturases have been identified [20-24]. In addition, a fatty acid reductase has been identified in *Bombyx mori *[25]. For understanding the evolution of moth sexual communication it is essential to know which genes are involved in the production of specific pheromone components and the variation in blend ratios.

The sex pheromone of *Heliothis virescens *(Fabricius 1777) (Lepidoptera: Noctuidae) is well-defined and consists of Z11-16:Ald as the major component, Z9-14:Ald as the secondary critical component, without which conspecific males are not attracted, and a few other minor components (16:Ald, Z7-16:Ald, Z9-16:Ald, Z11-16:OH), the roles of which roles are less clear [26-32]. We recently found geographic as well as temporal variation in this pheromone blend [33], which may be due to genetic variation.

The identification of genes that are expressed in the pheromone gland of *H. virescens *is not only useful for a fundamental understanding of moth sex pheromone evolution, but also from an applied perspective. *Heliothis virescens *is a major agricultural pest in a number of crops in North and South America. Pheromone traps are widely used in the US cotton belt to monitor *H. virescens *populations [34-37], and in Mexico [38]. The trap capture data have been used in many integrated pest management programs to determine if and when insecticide applications are needed. However, the extensive use of such traps may select for a shift in pheromone composition in local populations and thereby reduce trap efficiency [39]. Identifying the genes causing variation in pheromone signals can aid in delaying or even circumventing such adaptation.

We have taken two complementary approaches to identifying moth pheromone genes. One starts with the phenomenology of pheromone differences between different species, and attempts to find the genes responsible for these. We have investigated the genetic basis of sex pheromone variation within and between two closely related moth species, *H. virescens *(Hv) and *H. subflexa *(Hs) using quantitative trait locus (QTL) analysis [40,41]. We found 8 QTL that explained a significant proportion of the variance of 9 pheromone components. By considering how each QTL affects the overall blend, we generated a list of candidate genes that may underlie these QTL by linking the genomic regions to the biosynthetic pathway of the different sex pheromone components of the two species [40]. We have illlustrated this approach in the context of a recent review of the predominant pathways of Lepidoptera sex pheromone biosynthesis [42].

The other approach to identifying genes controlling the chemical communication system of moths starts with a large set identified on the basis of expression in the pheromone gland, and attempts to narrow this down to subsets that could be directly involved in biosynthesis and emission of the blend. Here we describe the construction and analysis of cDNA libraries made from Hv PG and associated tissues, which we compare to a) larval Hv libraries to identify genes that do not have an exclusive pheromone function, and b) PG libraries from other moth species to identify genes that might. To illustrate the context of these comparisons, we provide a brief description of PG structure.

**Figure 1:**
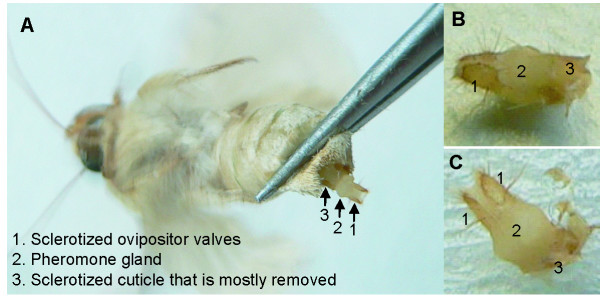
**Dissection of Hv sex pheromone gland for RNA extraction**. **A**. Gland was forced out by squeezing the abdomen with pincet (the gland is similary inflated when the female calls (Groot, Schal, Gould, Classen, pers. obs). **B**. Gland was cut at the scleritozed cuticle from the 8^th ^abdominal segment. **C**. Sclerotized cuticle was mostly removed before immersing gland in Trizol.

The pheromone gland is intimately associated with the ovipositor at the end of the adult female abdomen. The gland itself consists of a band-like single layer of epithelial cells that encircles the lower part of the eighth abdominal segment and the upper part of the ninth and last abdominal segment (Figure 1). These cells synthesize, store and release pheromone components in response to hormonal stimulation [8,9,19,43]. The outer surface of this band is covered with a thin cuticle densely packed with fine hairlike projections through which the pheromone is expelled [9]. The apical surfaces of the epithelial PG cells are elaborated into short microvilli pressing against the inner surface of this cuticle. When the abdomen is fully extended, the entire outer surface is exposed to the outside; when retracted, the layer is folded over itself and covered by the sclerotized upper half of the eighth abdominal segment [9]. The basal PG cell surfaces are underlain by a basement membrane separating the band of cells from the posterior end of the hemocoel cavity. Encircled by and passing through the band of glandular cells are the oviduct and the posterior section of the digestive tract, sheathed in muscle and connective tissue. The sclerotized end of the ninth abdominal segment is the ovipositor, which surrounds the ovipore and the anus.

To identify genes that could be involved in pheromone biosynthesis, we constructed normalized and non-normalized cDNA libraries of the pheromone gland plus ovipositor of *H. virescens*. In total we identified 8,310 gene objects. Comparing this database with a Hv larval tissue library, we found 6,435 gene objects that were unique to the PG libraries. We compared these to an EST collection from *Agrotis segetum *PG [44] and to a set of unpublished PG sequences from *Bombyx mori *deposited in GenBank. With the characterization of >8000 expressed gene objects and the comparisons between the different libraries, we can now list 86 candidate genes encoding classes of enzymes involved in the biosynthetic pathway of moth sex pheromone production. In addition, in the pheromone gland we found evidence for 27 genes that might be involved in pheromone perception and degradation, and a surprisingly high number (34) of antimicrobial effector genes involved in immune defense.

## Results

### Overall description of the sequences

The average insert size of the cDNAs of the Hv pheromone gland library that were cloned and sequenced was 1,100 bp, yielding a total of 6,554 ESTs, which after assembly resulted in a total of 2,692 contiguous sequences (contigs) and 1,799 singletons represented by a single EST (HvPGNoN; Table 1). After normalization, an additional 10,679 clones were sequenced, which resulted in an additional 6,772 putative gene objects (HvPGN). Combined, the non-normalized and normalized cDNA library (denoted as HvPG hereafter) resulted in a total of 2,082 contigs and 6,228 singletons. These sequences were subjected to a protein translated blastx search and a gene ontology (GO) analysis using Blast2GO [45]. 4,260 Sequences (52%) matched described sequences in Genbank (NR database; E-value cut-off of 10-3). Nearly half of the sequences (4,110) had no BLAST result (Table 1), indicating a high number of Lepidopteran or species-specific transcripts [46] or long UTRs without open reading frames. However, our method was based on directional cloning of full-length enriched cDNAs and 5'end-sequencing of the cDNAs. This strategy has potentially great advantages, as according to our findings, 5' UTRs in moths are generally much shorter on average (often < 100 bp; personal observation) than the 3' UTRs. For the majority of ESTs for which we could not obtain any hits to Genbank sequences we were still able to obtain open reading frames and InterPro scan results.

**Table 1:** Summary of *Heliothis virescens and Bombyx mori *cDNA libraries and the results of expressed sequence tag (EST) analysis

Name	Species	Tissue source	Av. insert size (bp)	No. sequences	No. contigs	No. singletons	Total no. gene objects	Gene objects with BLAST hits
HvPGnoN	*Heliothis virescens*	Pheromone gland	1100	6554	893	1799	2692	1871 (70%)
HvPGN	*Heliothis virescens*	Pheromone gland - normalized	950	10679	1324	5448	6772	3113 (46%)
HvPG	*Heliothis virescens*	Pheromone gland - combined	1000		2082	6228	8310	4260 (52%)
HvLN	*Heliothis virescens*	Mixed larval stages	1100	10511	1648	6174	7822	4302 (55%)
HvPG_minus_HvLN*	*Heliothis virescens*	Non-overlapping ESTs	NA	NA	NA	NA	6435	2691 (41%)

BmPG	*Bombyx mori*	Pheromone gland	NA	12296	1794	2153	3947	2749 (69%)

*See text for explanation

The average insert size of the cDNAs from mixed larval stages of *H. virescens *(the HvLN library) was 1,100 bp, similar to HvPG. The sequenced clones yielded a total of 10,511 sequences, which assembled to a total of 7,822 gene objects, 6,174 (79%) of which were singletons. Slightly more than half of these (4,302) showed a significant sequence similarity to sequences in the GenBank nr protein database, while the remaining 3,520 did not (Table 1).

The most highly expressed genes in the non-normalized portion of HvPG encoded proteins involved in general cellular homeostasis, like cell cytoskeleton and cellular organization (actins, tubulin), muscle proteins (myosin, tropomyosin), ribosomal proteins, and mitochondrial respiratory chain and ATP synthase proteins (see Additional file 1). Yet many of the top 250 highly expressed genes had no significant BLAST hit, which likely reflects the shallow genome coverage and annotation in the NCBI Insecta database.

### Comparisons of cDNA libraries

When we compared the EST dataset of HvPG to the ESTs generated from Hv larval tissue, 6,435 of the 8,310 sequences (i.e. 77%) were not found in HvLN. We will refer to this dataset as HvPG_minus_HvLN. Of these 6,435 sequences, only 41% (2,691) yielded high-score hits to public databases, while the majority (3,763, 59%) did not show any homology. The relatively high percentage of sequences with no significant homology to public databases indicates that many pheromone gland-specific genes have not been identified yet, similarly to what was found by Strandh et al. [44] in *Agrotis segetum*.

**Figure 2:**
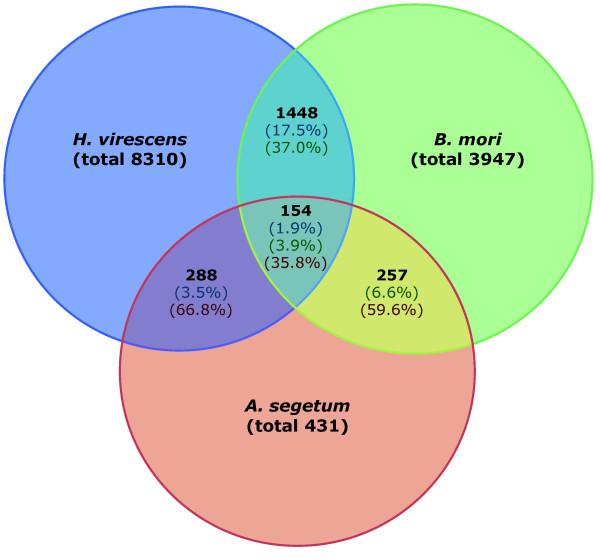
**Venn diagram showing the total number of contigs (in black) overlapping between the three pheromone gland EST databases**. Percentages are given of the relative number of overlapping contigs compared to the total number of contigs found in the pheromone gland (colors match the species colors).

Strandh et al. [44] constructed a cDNA library from the pheromone gland of *Agrotis segetum *and deposited 707 EST sequences in GenBank (accession numbers ES582156-ES584441). Assembling these sequences using the same procedure as described above resulted in a total of 431 contigs. We will refer to this dataset as AsPG. A total of 154 sequence homologies (best bidirectional hits) were found between this database and HvPG and BmPG (Figure 2).

From *Bombyx mori *we assembled a collection of sequences that from 12,296 ESTs that were deposited in Genbank (GenBank Acc: AV403746-AV404455, BP182009-BP184340, DC545768-DC550742, EL928418-EL930129) and identified as originating from the pheromone gland of *B. mori *(we refer to this dataset as BmPG). Assembling these sequences using the same procedure and threshold settings as for HvPG and HvLN resulted in a total of 3,947 gene objects, 2,153 (55%) of which were singletons. For all gene objects that we describe here as candidate genes for pheromone biosynthesis, we deposited sequences in the TSA section of Genbank (GenBank Acc: EZ407129-EZ407280). Of this dataset, 69% (2,749 sequences) had homology to sequences in the public database (NCBI nr). When comparing the sequences of BmPG with HvPG, we found homology between 1,448 sequences (best bidirectional hits) between the two datasets (see Figure 2).

### Assignment of putative gene functions using Gene Ontology

**Figure 3:**
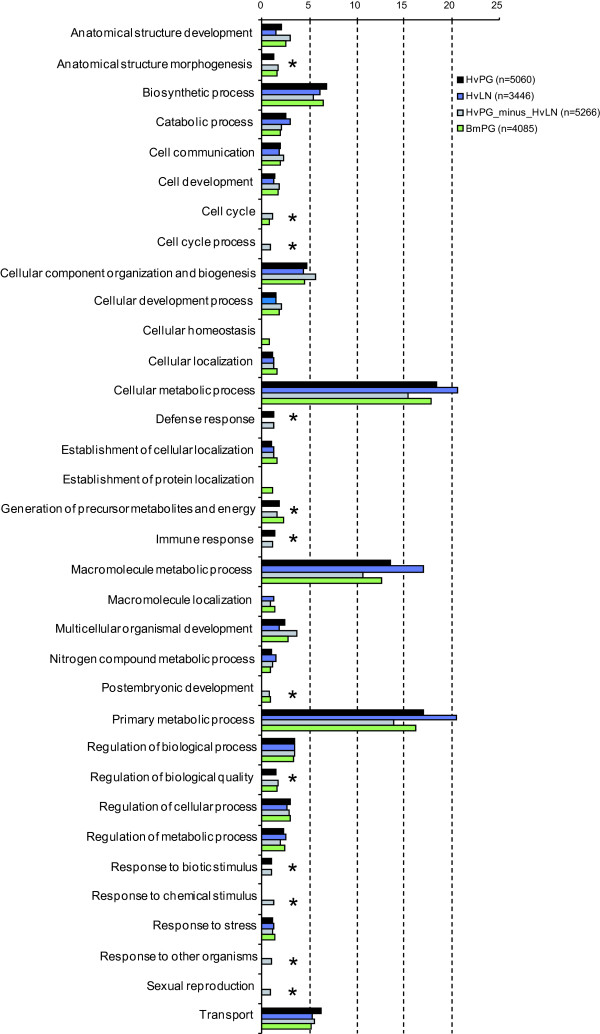
**All pie charts combined into a bar graph; overview of GO-level 3**. Note that one gene object can be classified into more than 1 class, therefore the total number of gene objects classified for Hv-PGN is not 2451 (2501 - 50), but 5060, indicating that on average one contig is classified into 2 classes. Asterisks denote absence in one of the libraries in the respective GO category.

For functional comparisons, all sequences were subjected to Gene Ontology (GO) analysis in Blast2GO, where we classified all gene objects in Biological Function level 3 (Figure 3). To minimize the number of classes with only few gene objects, we set the minimum number of gene objects (cut-off level) in a class to 2% of the total number of sequences that could be classified. In this comparison AsPG was excluded, because the AsPG EST dataset contained relatively few total sequences compared to HvPG, HvLN, HvPG_minus_HvLN, and BmPG.

Of the 4,260 sequences in the HvPG cDNA library with matches in the Genbank non-redundant (nr) protein database, 2,501 (59%) could be classified into GO-level 3, with each class containing at least 50 sequences (2% of 2501). Of the 4,302 HvLN sequences with high-score hits, 1,979 (46%) were classified into GO-level 3, with at least 41 sequences in each class. Of the 2,691 HvPG_minus_HvLN sequences, 1,929 (72%) were classified into GO-level 3. The classes containing at least 41 sequences are shown in Figure 3. Of the 2,749 BmPG sequences that showed homology to Genbank entries, 1,663 (60%) were classified into GO-level 3, with each class containing at least 36 sequences.

Figure 3 shows a total of 34 GO level-3 classes into which the gene objects were classified. Most occurred in Cellular metabolic process, Macromolecule metabolic process and Primary metabolic process (each with 10-20% of the total), followed by the classes Biosynthetic process, Cellular component organization and biogenesis, and Transport (each with about 5%). Three classes were absent in HvLN, but present in the other three libraries: Anatomical structure morphogenesis, Generation of precursor metabolites and energy, and Regulation of biological quality. The three classes Defense response, Immune responses and Response to biotic stimulus were only found in HvPG and HvPG_minus_HvLN, and thus did not occur (above the threshold level) in BmPG or in HvLN. The two classes, Cell cycle and Postembryonic development, were found only in HvPG_minus_HvLN and BmPG, while Cellular homeostasis and Establishment of protein localization were uniquely found in BmPG. Four classes were uniquely found in HvPG_minus_HvLN: Cell cycle process, Response to chemical stimulus, Response to other organisms, and Sexual reproduction.

The main contributor to the class of Immune responses was the class of antimicrobial peptides (AMPs). Insects produce a variety of AMPs with antibacterial and antifungal activity, including the insect defensins, cecropins, attacin-like proteins, lysozyme and proline-rich peptides [47-49]. In the HvPG we have identified a large number of AMPs, among which the cecropin gene family (10 genes), the gloverins (6 genes) and the moricin-like sequences (5 genes) are especially prominent (see Table 2). In addition to many newly identified AMP genes, in our pheromone gland library we were also able to identify all of the published antibacterial and antifungal proteins from *Heliothis virescens*, such as attacin [50], heliomicin [51], virescin (P83416), heliocin (P83427), lysozyme [52] and several cecropins (cecropins-A (P83413), -B (P83414), -C(P83415)).

**Table 2:** Genes encoding antimicrobial peptides involved in immune defense.

Gene	# gene objects found in HvPG	Contig numbers
Attacin	3	954, 1839, 4351
Cecropin	10	442, 443, 444*, 658, 850, 1852, 3420, 5571*, 5683, 6487*
Defensin	1	2179
Gloverin	6	501, 863, 864, 1201, 1370, 5807
Lebocin (heliocin*)	2	1198, 8170*
Anionic AMP	3	749, 751, 1013
Moricin-like (virescin*)	4	69, 1806, 1895*, 5291
Lysozyme	5	925, 3410, 4540, 4882, 5322

*identical to published *Heliothis virescens *AMPs (Genbank accession no. in brackets): cecropin-A (P83413), cecropin-B (P83414), cecropin-C (P83415), heliocin (P83427), virescin (P83416), lysozyme (AAD00078).

### Control of pheromone production and release

Pheromone production in the PG is stimulated by Pheromone Biosynthesis Activating Neuropeptide (PBAN) that is released from the suboesophagal ganglion in the brain to the hemolymph, after which it binds to the PBAN receptor in the membrane of the pheromone gland [43,53,54]. The PBAN receptor in the PG has been characterized as a G-protein-coupled receptor in *B. mori *by Hull et al. [53] (AB181298) and cloned from *H. virescens *by Kim et al. [55]. We found none of the three isoforms reported by that group (EU000525, EU000526, EU000527) in HvPG. EZ407266, occurring in HvPG but not HvLN, shows homology to a G-protein coupled receptor but is more similar to the diapause hormone receptor of *B. mori *(BAE93495). EZ407267 corresponds to a G-protein gamma subunit homologue, also found by Strandh et al. (AS12G05-D2), which they hypothesized to interact with the PBAN receptor.

**Figure 4:**
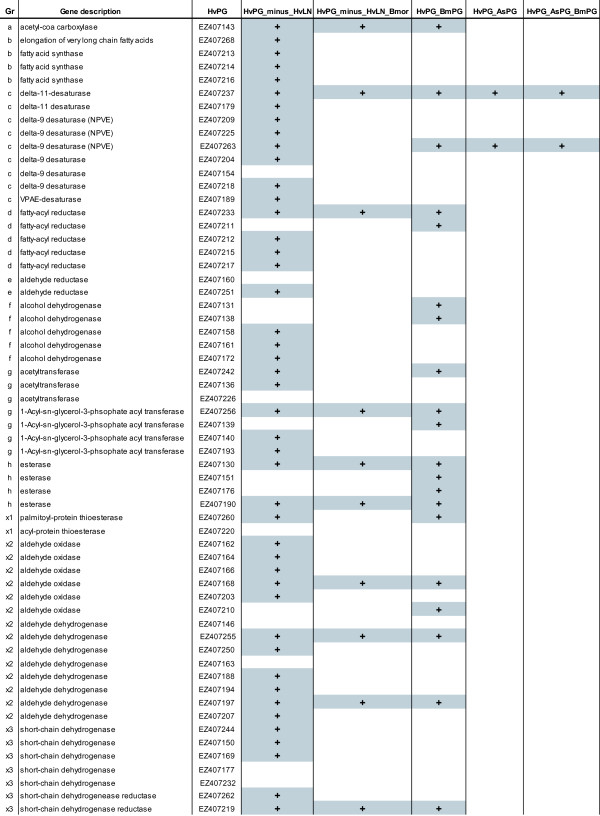
**Overlapping gene objects (in grey) possibly involved in the biosynthetic pathway of sex phermone production when comparing the different libraries (categories the same as in Figure 5)**. HvPG_minus_HvLN: ESTs in HvPG that were not found in HvLN. HvPG_minus_HvLN_BmPG: ESTs in HvPG that were not found in HvLN, but were found in the BmPG library. HvPG_BmPG: ESTs that were found both in HvPG and in BmPG. HvPG_AsPG: ESTs that were found both in HvPG and in AsPG. HvPG_BmPG_AsPG: ESTs that were found in HvPG, BmPG and AsPG.

Juvenile hormone has been shown to be an important regulator of pheromone induction in cockroaches [56], and bark beetles [57], but its role in the reproductive behavior in Lepidoptera has not yet been clearly defined [17]. In some species it likely influences the circadian release of PBAN [58], in others it may up-regulate the PBAN-receptor protein in the pheromone gland [59]. In HvPG (but not HvLN) we found two JH binding proteins, EZ407156 and EZ407196: the latter was also found in BmPG and AsPG (see Figure 4). Finding these sequences specifically in the pheromone gland suggests that they may play a role in the binding of PBAN to its receptor [59].

### Biosynthetic pathways of sex pheromone production

**Figure 5:**
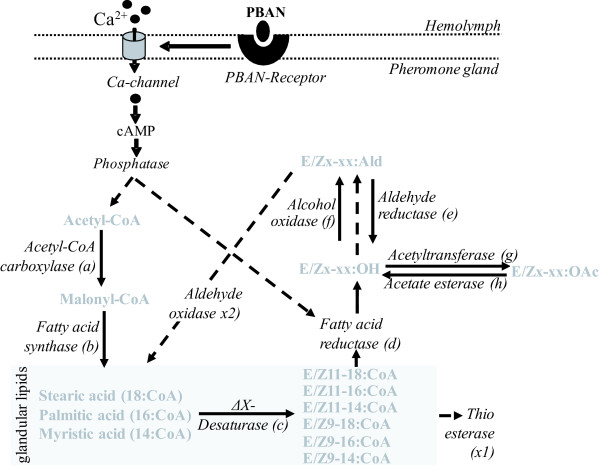
**Proposed biosynthetic pathway of sex pheromone production in female moths (adapted from **[14,17,25].

Considering the putative biosynthetic pathways of sex pheromone production in moths (Figure 5), we identified 70 candidate genes from the HvPG library that are likely to play a role (see Table 3 and Figure 4). Each enzyme in this pathway is categorized (a) through (h) to facilitate the comparisons between Table 2 Figures 5 and 4. Genes that have not been specifically described as being part of the pathways so far, but which may also be involved, are categorized as x1-x8. The list of contigs were compared against the other databases using reciprocal blast searches to identify which of the contigs are unique to HvPG (i.e. do not occur in HvLN; the HvPG_minus_HvLN database), and/or are also found in BmPG andAsPG (see Figure 4).

**Table 3:** Gene objects possibly involved in the biosynthetic pathway of pheromone production

Gene	# gene objects found in HvPG	GenBank Accession Numbers
(a) Acetyl CoA Carboxylase	1	EZ407143
(b) Fatty acid synthase	3	EZ407213, EZ407214, EZ407216
(b) Elongation of very long chain fatty acids	1	EZ407268
(c) Desaturases:		
d11-desaturase*	2	EZ407237, EZ407179
d9-desaturase (NPVE	3	EZ407263, EZ407209, EZ407225
d9-desaturase*	3	EZ407154, EZ407218, EZ407204
VPAE	1	EZ407189
(d) Fatty acyl reductase	5	EZ407233, EZ407211, EZ407212, EZ407215, EZ407217
(e) Aldehyde reductase	2	EZ407160, EZ407251
(f) Alcohol dehydrogenase (oxidoreductase)	5	EZ407131, EZ407138, EZ407158, EZ407161, EZ407172
(g) Acetyltransferase	3	EZ407136, EZ407242, EZ407226
(g) 1-Acyl-sn-glycerol-3-phsophate acyl transferase	4	EZ407193, EZ407256, EZ407139, EZ407140
(h) Esterase	4	EZ407130, EZ407151, EZ407176, EZ407190
(i) Lipase (neutral)	5	EZ407271, EZ407272, EZ407274, EZ407275, EZ407276
(i) Lipase (acidic)	5	EZ407273, EZ407277, EZ407278, EZ407279, EZ407280
(x1) Palmitoyl-protein thioesterase	1	EZ407260
(x1) Acyl-protein thioesterase	1	EZ407220
(x2) Aldehyde oxidase	6	EZ407162, EZ407164, EZ407166, EZ407168, EZ407203, EZ407210
(x2) Aldehyde dehydrogenase	8	EZ407163, EZ407194, EZ407146, EZ407255, EZ407250, EZ407188, EZ407197, EZ407207
(x3) Short-chain dehydrogenase	5	EZ407232, EZ407244, EZ407150, EZ407169, EZ407177
(x3) Short-chain dehydrogenase reductase	2	EZ407262, EZ407219
(x4) Acyl-CoA oxidase	5	EZ407175, EZ407178, EZ407208, EZ407221, EZ407265b
(x4) Acyl-CoA dehydrogenase	1	GU205155
(x5) Enoyl-CoA hydratase	3	GU205156, GU205159, GU205160
(x6) 3-Hydroxyacyl-CoA dehydrogenase	2	GU205156, GU205162
(x7) Enoyl-CoA isomerase	1	GU205161
(x8) 3-Ketoacyl-CoA thiolase	4	EZ407147, EZ407229, EZ407242, GU205158

*Homologous sequences were found in Strandh et al (Δ11-desaturase &ES583599, Δ9-desaturase &ES583724)

#### (a) Acetyl CoA carboxylase (EC:6.4.1.2)

**Figure 6:**
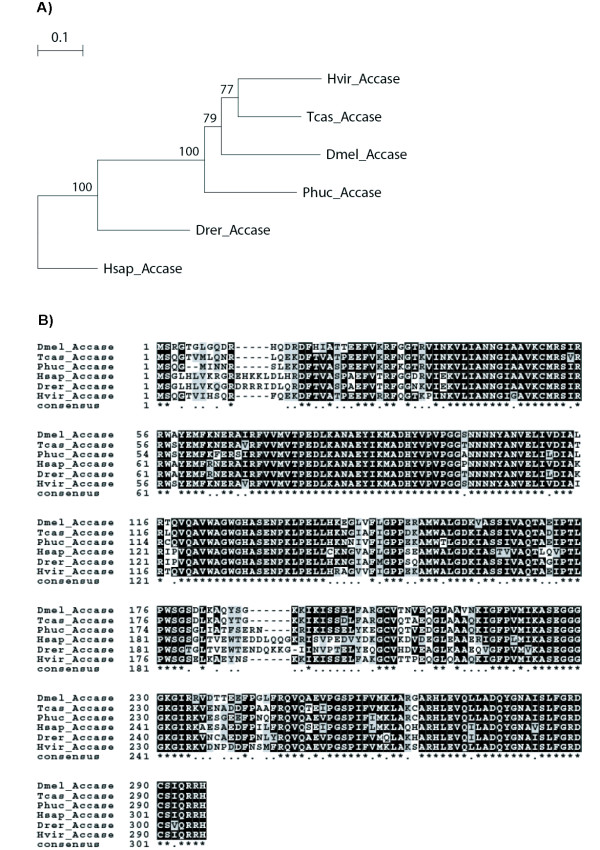
**Gene phylogeny and sequence similarity of Accase protein sequences**. **A**. Neighbor-joining (NJ) consensus tree of ACCase sequences from *Heliothis virescens *(Hvir), *Tribolium castaneum *(Tcas; XP_969851), *Pediculus humanus corporis *(Phuc; XP_002429216), *Drosophila melanogaster *(Dmel; NP_610342), *Danio rerio *(Drer; XP_001919815) and *Homo sapiens *(Hsap; AJ575431). Bootstrap values from NJ analyses are shown as percentages. **B**. MAFFT alignment with part of Accase proteins listed in the phylogeny. Identical amino acids are shaded in black and depicted by an asterisc, conserved amino acids are shaded in grey and depicted by a dot in the consensus sequence.

EZ407143 in HvPG matched sequences in the public database described as Acetyl-CoA carboxylase (ACCase). This enzyme catalyzes the ATP-dependent carboxylation of acetyl-CoA to malonyl-CoA in the rate-limiting step of long chain fatty acid biosynthesis [60]. A search in NCBI for "Acetyl CoA Carboxylase AND Lepidoptera" returned a sequence from *Heliothis virescens *(CS239503, Sequence 1 from Patent EP1607477-A 1) which is identical to EZ407143. This contig also showed high sequence similarity to ACCases described in other insects (*Drosophila melanogaster, D. pseudoobscura, Nasonia vitripennis, Apis mellifera *and the moth *Cydia pomonella*) and mammals. An alignment of the ACCase sequences from insects and vertebrates shows an overall very high sequence similarity (Figure 6). Amino acid sequence identity between *H. virescens *and *H. sapiens *is 72%. ACCase (EZ407143) was not found in HvLN; a similar sequence was present in BmPG, but not in the much smaller AsPG.

#### (b) Fatty acid synthase (EC:2.3.1.85)

Malonyl-CoA, acetyl-CoA, and NADPH are utilized in fatty acid synthesis by the multifunctional enzyme Fatty Acid Synthase (FAS). Labeling studies with acetate demonstrated that the principal end products of FAS in most lepidopteran PGs are palmitic acid (16:0) and stearic acid (18:0) [61-63]. We found three contigs (see Table 3) that showed high sequence similarity with described insect FAS in Genbank (in *Drosophila*, *Aedes*, and *Nasonia*) as well as FAS of chicken and *Mus musculus*. In addition, EZ407268 was described in Blast2Go as 'elongation of very long chain fatty acids' (based on the top BLAST hits of the nr database entries). All four contigs were absent from HvLN. We did not find any significant hits of these four HvPG sequences to any of the BmPG or AsPG ESTs using reciprocal blast searches, which again is most likely due to the lower number of transcripts identified in these libraries.

#### (c) Desaturases (EC:1.14.19)

In pheromone biosynthesis, double bonds are introduced into the fatty acid chain by a variety of desaturases, which have been classified into groups based on signature motifs, and extensively characterized biochemically [21]. Two contigs were identified as putative Δ11-desaturase, while 7 were identified as putative Δ9-desaturases and EZ407189 was identified as a desaturase with the signature motif of VPAE (Table 3). It is not surprising to find a number of Δ9-desaturases, because they also occur commonly in animal and fungal tissues [22]. However, EZ407154 was the only Δ9-desaturase that was also found in the transcriptome of HvLN, the other sequences were only found in HvPG. Two groups of Δ9-desaturases have been identified and characterized in pheromone glands of moth species: one with a substrate preference of C_16 _> C_18 _(called NPVE), and the other with a substrate preference of C_18 _> C_16 _(referred to as KPSE) [21,24]. Three contigs resembled predicted amino acid sequences of characterized desaturase genes displaying an NPVE signature motif, while the other 4 contigs resembled Δ9-desaturase sequences in other insects but did not contain the signature motif of either NPVE or KPSE. Specifically, EZ407263 and EZ407218 most closely resembled Δ9-desaturase in *H. zea *(AAF81790), EZ407204 mostly resembled the Δ9-desaturase of *Lampronia capitella *(ABX71627), while EZ407154 mostly resembled Δ9-desaturase AAF81788 of *H. zea*. One of the Δ11-desaturases (EZ407237) and one of the Δ9-desaturases (EZ407263) were found in BmPG and AsPG as well and may thus be involved in the biosynthetic pathway of pheromone production in all of these species. For example, EZ407237 resembles a sequence from AsPG (AS12G02_D9), which was 84-fold upregulated compared to the As-body library [64].

#### (d) Fatty acyl reductase (EC:1.2.1.-)

**Figure 7:**
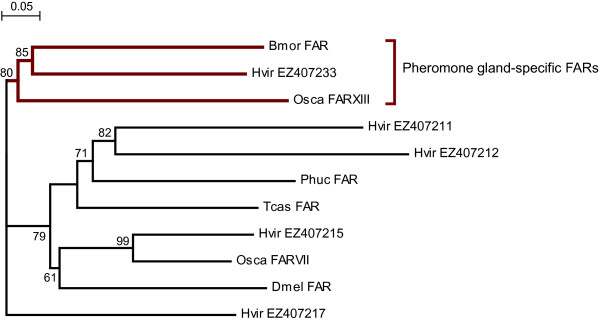
**Gene phylogeny of FAR protein sequences**. Neighbor-joining (NJ) consensus tree of FAR sequences from *Heliothis virescens *(Hvir), *Bombyx mori *(Bmor; NP_001036967), *Ostrinia scapulalis *(Osca; ACJ06514), *Tribolium castaneum *(Tcas; XP_967757), *Pediculus humanus corporis *(Phuc; XP_002428142), and *Drosophila melanogaster *(Dmel; AAF46099). Bootstrap values from NJ analyses are shown as percentages of a total of 1000 bootstrap runs.

There is evidence for two routes of aldehyde pheromone biosynthesis in moths. The fatty acyl CoA pheromone precursor may be reduced to the corresponding alcohol by an alcohol-generating Fatty Acyl Reductase (FAR) and then oxidized to the corresponding aldehyde by an alcohol oxidase, i.e. dehydrogenase [17]. Alternatively, fatty acyl CoA may be reduced directly to aldehydes by aldehyde-generating FARs. We found five contigs resembling FAR, only one of which (EZ407211) was also found in HvLN (Figure 4). Phylogenetic analysis of the FAR sequences identified in *H. virescens *and of a subset of fatty-acyl-reductases in Lepidoptera and other insects (Figure 7) indicate that the *H. virescens *EZ407233 can be grouped with FARs from pheromone glands of *B. mori *and *Ostrinia scapulalis*. The alcohol-generating FAR from *B. mori *was shown by functional expression to produce the pheromone bombykol from its precursor [25]. The FAR-XIII gene of *O. scapulalis *was the only one of 13 specifically expressed in the pheromone gland [65]. EZ407233 is specifically expressed in the pheromone gland as well (Groot and Barthel, unpubl. res).

#### (e) Aldehyde reductase and (f) Alcohol oxidase (EC:1.1.1.2)

Whether aldehyde reductases first produce aldehydes which are then converted to alcohols, or vice versa, is very difficult to prove, because aldehyde reductases can also catalyze the reduction of the fatty aldehyde to the alcohol, so alcohols and not aldehydes are the major products [66]. The reverse reaction is catalyzed through alcohol oxidases, and both enzymes are more generally described as alcohol dehydrogenases. The systematic name of this group of enzymes is alcohol:NADP+ oxidoreductase, oxidizing alcohols using NAPD+ (alcohol + NADP(+) <=> aldehyde + NADPH). Some enzymes in this group oxidize only primary alcohols, while others act also on secondary alcohols. This group of enzymes may be identical to glucuronate reductase (EC 1.1.1.19), mevaldate reductase (EC 1.1.1.33) and lactaldehyde reductase (EC 1.1.1.55). Two contigs were named aldehyde reductase in Blast2GO, one of which (EZ407251) did not occur in the HvLN transcriptome, while five contigs were named alcohol dehydrogenase, three of which were uniquely present in the HvPG transcriptome (see Figure 4). None of these sequences were found in BmPG or AsPG.

#### (g) Acetyltransferase (EC:2.3.1.16)

In general, synthesis of phospholipids can occur *de novo *or via remodeling of the existing phospholipids, and the biosynthesis of triglycerides (a form of energy storage in cells) is an end product of these pathways [19]. In animals members of the 1-acyl-*sn*-glycero-3-phosphate acyltransferase family (AGPATs; EC:2.3.1.51) have been shown to transfer unsaturated fatty acyl groups. Several AGPATs acylate lysophosphatidic acid (LPA) at the carbon-2 position to produce phosphatidic acid (PA). These enzymes are involved in phospholipid and triglyceride synthesis through an evolutionary conserved process involving serial acylations of glycerol-3-phosphate (Acyl-CoA + 1-acyl-sn-glycerol 3-phosphate <=> CoA + 1,2-diacyl-sn-glycerol 3phosphate; http://www.expasy.org/enzyme/2.3.1.51). We found four distinct sequences similar to AGPATs, of which only one was also found in the transcriptome of HvLN, whereas the others were only found in the HvPG library. One of the sequences that was present in HvPG but absent in HvLN (EZ407256) was found in BmPG as well. In addition, three distinct putative acetyl-CoA-acyltransferase sequences were found in HvPG, two of which were not found in HvLN. This functional class of enzymes potentially converts fatty alcohols to acetate esters in pheromone glands and has been biochemically characterized in *C. fumiferana *[15] and *Argyrotaenia velutinana *[67]. Remarkably, no acetyltransferase genes have been cloned from moth pheromone glands so far, although acetate esters are common pheromone components.

#### (h) Acetate (acetyl) esterase (EC:3.1)

Esterases are hydrolases, and hydrolysis of esters occurs during pheromone synthesis and degradation [68-70]. Acetate esterases in pheromone glands have been shown to be active biochemically in *C. fumiferana *[15], *Hydraecia micacea, H. virescens *and *H. subflexa *[71]. In *H. subflexa *three acetates are components of the pheromone blend [72-75], which render it unattractive to *H. virescens *males [3,32]. Acetates have never been found in the pheromone gland of Hv, and Teal and Tumlinson [71] suggested that acetate esterase converts the acetates into alcohols as rapidly as they are produced in *H. virescens*, but not in *H. subflexa*. We found four esterases in HvPG, two of which were not found in HvLN. All four contigs were also found in BmPG.

#### (i) Lipase (EC 3.1.1.3)

Some of the fatty acids incorporated into pheromones may be liberated from pre-existing phospholipids and triglycerides by cleavage by lipases, to supplement fatty acid synthesis de novo [19]. Most insect lipases fall into two categories, acidic (so named due to similarity to mammalian gastric lipases which function at acidic pH) and neutral (similar to mammalian pancreatic lipases) [76]. We found five neutral lipases (EZ407271, EZ407272, EZ407274, EZ407275, and EZ407276), and five acidic lipases (EZ407273, EZ407277, EZ407278, EZ407279, and EZ407280) in HvPG. Horne et al. [76] suggested that the lipase encoded by AV403884, an EST from *B. mori *pheromone gland was involved in liberation of fatty acids for pheromone precursors; this is most similar to our EZ407279 from HvPG.

#### (x1) Acyl-CoA Thioesterasesl Long-chain fatty-acyl-CoA hydrolase (EC:3.1.2.2)

Acyl-CoA thioesterases (also known as acyl-CoA hydrolases) are a group of enzymes that hydrolyze CoA esters to free CoA and carboxylic acids. Substrates can be a range of diverse molecules such as acyl-CoAs (which can either be saturated, unsaturated, or branched-chain), CoA esters of prostaglandins, bile acid CoAs etc. Acyl-CoA thioesterases have important functions in lipid metabolism and in regulating the levels of free CoA and various CoA esters in the cell, and have been found in the cytosol, mitochondria, and peroxisomes [77,78] The thioesterase sequences we identified were most similar to palmitoyl-protein thioesterase (EZ407260) and acyl-protein thioesterase (EZ407220). EZ407260 was not found in HvLN, while EZ407220 was found in the HvLN as well as in BmPG (see Figure 4).

#### (x2) Aldehyde oxidase/dehydrogenase (EC:1.2.1.3)

This group of enzymes catalyze the oxidation of aldehydes to carboxylic acids. Therefore they may also be involved in the synthesis or degradation of pheromone compounds in the pheromone gland, specifically in the conversion of aldehydes to carboxylic acids (stearic acid and/or palmitic acid) (Figure 5). A total of 14 distinct sequences that were categorized as aldehyde dehydrogenase were present in HvPG, 11 of which were not found in HvLN. Four of these sequences were also found in BmPG.

#### (x3) Short-chain alcohol dehydrogenase/reductase(EC:1.1.1.1)

The short-chain dehydrogenases/reductases family (SDR) is a very large family of enzymes, most of which are known to be NAD(P)- dependent oxidoreductases. This superfamily consists of a phylogenetically related group of enzymes that act on substrates as diverse as steroids, fatty acids, sugars, aromatic hydrocarbons, antibiotics, and compounds involved in nitrogen metabolism [79]. The *Drosophila *alcohol dehydrogenase belongs to this group of enzymes, and as it was the first member of this family to be characterized, the SDR family used to be called 'insect-type', or 'short-chain' alcohol dehydrogenases. Since a number of dehydrogenases or reductases are likely to be involved in the moth pheromone biosynthetic pathway (as described above) we have included this generic group of enzymes in our list of candidate genes (Figure 4). We found a total of 7 distinct sequences in HvPG, two of which were not unique to this dataset, i.e. EZ407177 and EZ407232. One of the SDR sequences, EZ407219, was also found in BmPG.

#### (x4) Acyl-CoA oxidase (EC:1.3.3.6), Acyl-CoA dehydrogenase (EC: 1.3.99.3)

Acyl-CoA oxidases (in peroxisomes) and acyl-CoA dehydrogenases (in mitochondria) are involved in the pathway responsible for lipid catabolism and catalyse the first step in fatty acid β-oxidation by forming a *trans-α,β *double bond. Apart from general lipid catabolism, acyl-CoA oxidases are involved in a larger number of pathways and cellular processes, including the biosynthesis of plant hormones, the PPAR signaling pathway, and also the biosynthesis of unsaturated fatty acids. We found in total five sequences resembling acyl-CoA oxidase, four of which were present in HvPG but not in HvLN (Figure 4). In addition, one mitochondrial acyl-CoA dehydrogenase was found. Since enzymes of the β-oxidation cycle act on CoA derivatives with 8 to18 carbon chain length fatty acids, they may be active in pheromone production or degradation as well.

#### (x5) Enoyl-CoA hydratase (EC: 4.2.1.17, EC: 4.2.1.74)

This enzyme carries out the second step in β-oxidation of fatty acids, the hydration of the *trans-α,β *double bond. Two were found in HvPG, GU205159 and GU205160. This enzyme activity is also represented by GU205156, corresponding to the α-subunit of the mitochondrial fatty-acid β-oxidation complex, which is a bifunctional protein also possessing 3-hydroxyacyl-CoA dehydrogenase activity.

#### (x6) 3-Hydroxyacyl-CoA dehydrogenase (EC: 1.1.1.35, EC: 1.1.1.211)

This enzyme performs the third step in β-oxidation of fatty acids, the NAD+ -dependent dehydrogenation of the β-hydroxyacyl CoA to form the β-ketoacyl-CoA. In HvPG this is represented by GU205162, and also by the bifunctional GU205156.

#### (x7) Enoyl-CoA isomerase (EC: 5.3.3.8)

This enzme assists in the β-oxidation of unsaturated fatty acids, transposing a C = C double bond so that subsequent steps of β-oxidation can be carried out. One representative, GU205161, was found in HvPG.

#### (x8) 3-Ketoacyl-CoA thiolase (EC: 2.3.1.16)

This enzyme performs the fourth and final step in the β-oxidation cycle, cleavage of a C-C bond to form acetyl-CoA and a new acyl-CoA with two fewer carbon atoms than the previous one. Four representatives, including one corresponding to the β-subunit of the mitochondrial fatty-acid β-oxidation complex (GU205158), were found in HvPG.

Strandh et al. [44] also described a number of sequences in the *Agrotis segetum *pheromone gland EST library that were considered as candidate genes putatively involved in pheromone production (their Table 4). Based on the similarity in gene descriptions of homologous sequences (best BLAST hits) found in Genbank, ten of these were potentially similar to sequences we have identified in the *Heliothis *libraries. To determine whether these sequences overlapping by their gene descriptions are indeed the same, we downloaded their candidate gene sequence files from Genbank and compared them with sequences from our libraries using SeqTools 8.4.042 (http://www.seqtools.dk, 2002-2008 S.W. Rasmussen). Two desaturase contigs in AsPG were also present in HvPG (see Table 3). Also, the AsPGcontig that showed homology to "Acyl-CoA binding protein homologue" (ES582331) was found in HvPG (EZ407246), as well as the JH binding protein (ES583149) which was homologous to EZ407196. The other contigs with homologies to Enoyl CoA hydratase (ES583704) and "Similar to acetyl CoA acetyltransferase precursor" (ES583111) were not found in HvPG.

**Table 4:** Gene objects likely involved in pheromone perception and/or degradation

Gene	# gene objects found in HvPG	GenBank Accession Numbers
Odorant binding protein*	4	EZ407182, EZ407186, EZ407195, EZ407200
Chemosensory protein*	16	EZ407243, EZ407247, EZ407249, EZ407254, EZ407257, EZ407261, EZ407137, EZ407165, EZ407129, EZ407157, EZ407173, EZ407270, EZ407201, EZ407227, EZ407230, EZ407231
Pheromone binding protein	2	EZ407264, EZ407174
Antennal binding protein	3	EZ407240, EZ407238, EZ407149
Chemosensory receptor 12	1	EZ407205
Odorant-degrading enzyme	1	EZ407198

*Homologous sequences were found in Strandh et al (OBP &; ES582180, Chem. protein &ES584018 and ES583800)

### Pheromone perception and degradation

**Figure 8:**
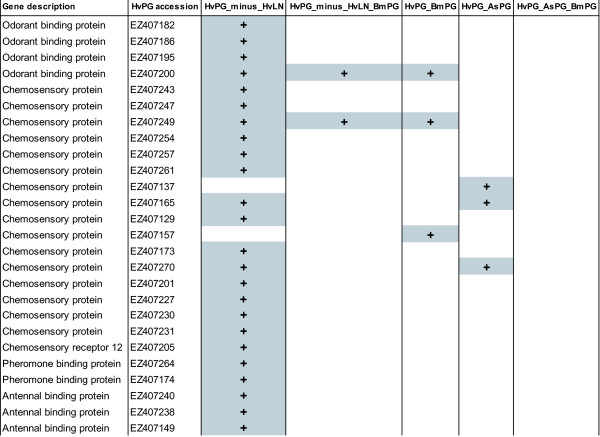
**Overlapping gene objects (in grey) involved in pheromone perception and/or degradation when comparing the different libraries (see Figure 4 for explanation of the headings)**.

Candidates involved in pheromone perception and/or degradation include odorant binding proteins OBPs), chemosensory proteins (CSPs), pheromone binding proteins (PBPs), antennal binding proteins (ABPs), chemosensory receptors, and odorant degrading enzymes (Table 4 and Figure 8). Most of these have been identified in antennae of adult males [16,80-87]. In many insect species, OBPs are a diverse gene family that encode proteins thought to function as molecular chaperones by binding pheromones and semiochemicals and transporting them through the aqueous lymph of insect sensilla to the olfactory receptors (ORs). Compared with OBPs, CSPs are expressed more broadly in various insect tissues. In the silkworm *Bombyx mori*, the OBPs are subdivided into three main subfamilies; pheromone-binding proteins (PBPs), general odorant-binding proteins (GOBPs) and antennal-binding proteins (ABPs) [88].

**Figure 9:**
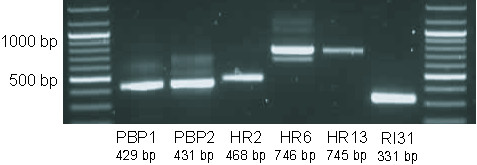
**PCR products using the primers described by Widmayer et al. (2009) on the normalized and non-normalized cDNA pool of the Hv pheromone gland**. Abbreviations used as in Widmayer et al: PBP: Pheromone Binding Protein; HR: Heliothis Chemosensory Receptor; RL: Ribosomal Protein.

Detailed anatomical studies of moth pheromone glands have not revealed similar sensory structures [8,9]. However, recently Widmayer et al. [89] detected the pheromone receptors HR2, HR6 and HR13 by RT-PCR in tissue consisting of the ovipositor tip and the PG. They subsequently identified a few sensillae expressing one of these pheromone receptors on the ovipositor, i.e. the sclerotized cuticle on the lateral lobes of the tip of the ninth abdominal segment. These pheromone receptors, as well as the pheromone binding proteins (PBP1 and PBP2) found by Widmayer et al. [89] were not present in HvPG. However, we could find these products after amplification with the gene-specific primers (see Figure 9).

The odorant binding proteins that we did find in HvPG, all were present in HvPG and absent in HvLN, indicating that these proteins are pheromone gland specific as well. One of these sequences coding for odorant binding proteins was also found in BmPG (EZ407200). Another major group of genes that we found in the pheromone gland library were 16 sequences that were listed as chemosensory proteins. Of the chemosensory proteins that we found in HvPG, only two also occurred in HvLN, one of which was found in BmPG (EZ407157) and one in AsPG (EZ407137). Of the 14 chemosensory proteins that were only found in HvPG and not in HvLN, one was found in BmPG (EZ407249), while EZ407165 and EZ407270 were also present in AgPG.

## Discussion

Our strategy of analyzing both non-normalized and normalized libraries of the same tissues allowed us to combine advantages of both; frequency counts of the most abundant transcripts in the former provided a "digital Northern", and reduction of these in the latter greatly increased the discovery rate of genes with lower expression levels.

The normalization resulted in the reduction of many over-abundant transcripts detected as strong bands in the non-normalized total cDNA, thus drastically increasing the number of new gene objects identified per sequencing run. The sequencing and assembly into 8310 contigs from the sex pheromone gland of *H. virescens *has revealed that this tissue is more complex than we envisioned beforehand. This is likely at least partly due to the fact that the pheromone gland tissue is intimately associated with the ovipositor, the oviduct and the last part of the digestive tract, as described in detail in the Introduction.

The main objective of this study was to identify a list of candidate genes that are likely to be involved in the biosynthetic pathway of moth sex pheromone production. By comparing our extensive database of the pheromone gland transcriptome of *H. virescens *to that of all 10,511 sequences identified from larval tissues of the same moth, as well as to the sequences identified from the pheromone gland of *B. mori *and *A. segetum*, we identified a total of 70 candidate genes that included all enzyme categories that have been biochemically found to be involved in the biosynthetic pathway of sex pheromone production in moths [14,17] (Figure 5). The fact that we found relatively few overlapping sequences in the different pheromone gland databases may either be due to unsaturated coverage or to highly diverged gene expression patterns between species leading to the species-specific pheromone blends.

The finding of a sequence homologous to the PBAN receptor in HvPG as well as in HvLN support the finding of Rafaeli et al. [54] who found the PBAN receptor in *H. armigera *also to be present in the brain, thoracic ganglion and ventral nerve cord, i.e. in neural tissues, suggesting that these receptors may represent a neurotransmitter-like function [54]. The fact that we and Strandh et al. [44] found an unrelated G-protein coupled receptor indicates that additional receptors are present in the pheromone gland and/or ovipositor than just the PBAN receptor.

Even though the enzymes that are possibly involved in the biosynthetic pathway of pheromone production do not necessarily have to be only present or active in the pheromone gland, if we assume that the contigs that are specifically found in HvPG and not in HvLN are more likely to be candidate genes for this pathway than contigs that were found in both datasets, then EZ407233 is the most likely candidate involved in pheromone production in Hv. This is because a) it is mostly expressed in the pheromone gland in comparison to the body (Groot and Barthel, unpubl. res.), and b) it is the FAR that most closely resembles the FAR of *B. mori *that has been found to be involved in the biosynthetic pathway [25] as well as the FARXIII of *Ostrinia scapulalis *that that was exclusively expressed in the pheromone gland [65] (see Figure 7).

As for the desaturases, in addition to the clearly defined Δ9- and Δ11-desaturases that we found in the pheromone gland transcriptome, we also identified a contig that mostly resembled a desaturase that has been named VPAE, after its signature motif [21]. Even though no function has been determined for this desaturase or others with the signature motif GATD, QPGE, and KPVE, they are also regularly found in sex pheromone glands, and codon-based likelihood analyses indicate strong purifying selection, so that these lineages are most likely protein coding and functional as well [21].

Having identified candidate genes of all categories that have been biochemically shown to be involved in the biosynthetic pathway of moth pheromone production opens the possibility to characterize more enzymes than only desaturases and fatty acyl reductases, which will certainly increase our understanding of how sex pheromones may evolve. Especially the acetate esterases are most likely to be found in pheromone glands of many moth species, as a large number of species have acetate esters as pheromone components. Identification of variation in this enzyme may shed light on the diversification of pheromone components in the different species. The molecular characterization of the additional enzymes involved in the biosynthetic pathway of moth pheromone production can also help to elucidate specific paths in the biosynthetic pathway. For example, the question of whether aldehyde reductases first produce aldehydes which are then converted to alcohols, or vice versa, may be solved by knocking down specific aldehyde reductases.

The diversity of antimicrobial peptides (AMPs) identified in the pheromone gland library of *Heliothis virescens *is both impressive and surprising (see Table 2). Since the first discovery of AMPs in insects [90], several hundred of these peptides with antifungal and/or antibacterial activity have been identified from diverse insects and many vertebrates. However, the *in vivo *function in insects of most members of this large and very diverse group of proteins still remains unclear. Although they lack an adaptive immune system, insects possess an innate immune system that recognizes and destroys intruding microorganisms. Parasites and pathogens can represent extremely powerful selective forces because of their ability to evolve rapidly. The resulting diversity of infectious agents and host immune-suppressive factors exerts strong selection pressures and favors hosts with a large repertoire of defense responses, including effector molecules with direct defense activity, among which AMPs and peptide families such as the defensins are prominent [91].

The identification of the vast amounts of AMPs in the *Heliothis virescens *pheromone gland and associated tissue is even more surprising given that Shelby et al. [92] have only identified a single AMP-like transcript in a survey of the immune-induced hemocyte transcriptome of *H. virescens*, despite the bacterial elicitation of the hemocytes in the construction of the cDNA library. However, the majority of AMPs circulating in the larval hemolymph seems to be produced in the fat body and other tissues, and not in the hemocytes themselves. In our pheromone gland library we have not only identified all of the published antibacterial and antifungal proteins from *Heliothis*, such as attacin [50], heliomicin [51], and lysozyme [52] but also a wide range of additional AMPs from these and other gene families, including a large variety of gloverins and cecropins (see Table 2). Possibly, this large variety is due to the fact that the gland is everted with the ovipositor to the outside when a female moth is calling, and thus exposed to the external environment.

The sequences found in *Bombyx mori *pheromone gland contained a much smaller number of gene objects that were classified as immune defense-related. This may be due to the fact that we extracted RNA from the gland with the ovipositor, similar to the procedure of Strandh et al. [44], as the pheromone gland is tightly connected and completely surrounding the ovipositor in Hv [9]. Since the pheromone gland of *B. mori *is much bigger, it is possible to dissect the gland from the ovipositor and care was taken to extract from the pheromone gland only (K. Mita, personal communication). However, the much smaller number of immune defense gene objects in *B. mori *may also reflect the fact that this moth is the oldest domesticated animal (since ~10,000 years), with one of the result being that adults are no longer able to fly. Probably under these domesticated conditions the environmental pathogen pressure against which the gland should be defended is absent as well, which may have caused the immune defense and immune response genes to be either not expressed or at a much lower level and with less complexity. An alternative explanation would be that in *B. mori *the steady-state level of immune defense is much lower as compared to non-domesticated Lepidopteran species. The steady-state immune defense levels in response to even non-pathogenic bacteria-feeding in larvae of herbivorous Lepidoptera has recently been shown to be higher than expected [93]. In the flesh fly, *Sarcophaga peregrina*, it was observed that mRNA of antibacterial genes accumulated even in naïve insects during different developmental stages [94].

Lastly, finding a total of 26 sequences that are likely to be involved in pheromone perception (see Table 4) strongly suggests that females may perceive pheromone compounds via their ovipositor and/or pheromone gland. Having chemical and/or pheromone receptors on and near the pheromone gland suggests that there may be a feedback loop of the chemosensory environment and the pheromone production [89]. Preliminary studies on phenotypic plasticity in the pheromone production of the closely related moth species *H. subflexa *indeed suggest that the pheromone composition differs depending on the environment in where the females developed (Groot, Staudacher and Claβen, unpubl. res.).

## Conclusion

We have generated an extensive list of candidate genes that may be involved in the pheromone biosynthesis, perception and/or degradation and which occur specifically in female sex pheromone glands. Subsequent evaluation of these candidates will follow two independent approaches. A correlation with active pheromone biosynthesis will be tested by determining which of these genes are differentially up- or downregulated in pheromone glands that are actively producing pheromone (i.e. in the scotophase or upon injection with PBAN; see [95]. In addition, a correlation with observed intra- and interspecific genetic variation in pheromone component ratios will be explored by mapping candidate genes onto our QTL map, generated by Sheck et al. [41] and Groot et al. [40]. Those genes exhibiting both correlations will be excellent candidates for further exploration by functional expression and RNAi technology.

## Methods

### Insects

*Heliothis virescens *eggs were collected in Clayton, NC, in 2005 and reared on artificial diet [96] in the laboratory of ATG at NCSU since then, under 27°C, 50-70% RH and 14:10 L:D light cycle. Larvae and pupae were sent to Jena in July 2007. Pupae were sexed and females and males were kept separately. The pupae were checked daily for emergence, so that adults were aged ± 12 h.

### Gland extractions

Pheromone glands were extracted from 3-5 day old females. Sixteen glands were extracted from females in the photophase (presumably not producing pheromone), and 16 glands were dissected from females that had been injected with Pheromone Biosynthesis Activating Neuropeptide (PBAN) 1-2 hours prior to extraction (see Groot et al. 2005). In summary, a stock solution of *Hez*-PBAN (Peninsula Laboratories, San Carlos, CA) (200 pmol/*μ*l in 50% methanol and 1 N HCl) was diluted in saline (PBS) to 3.75 pmol/*μ*l. Females were injected during the photophase with 7.5 pmol PBAN in 2 *μ*l, using a 10 *μ*l syringe (Hamilton, Reno, NV) with a 31 gauge needle that was inserted ventrally between the 8th and the 9th abdominal segments. One-two hr after injection, the pheromone glands were dissected (see Figure 1), placed immediately in Trizol and frozen to -80°C before homogenization and total RNA extraction. As can be seen in Figure 1, the complete intact gland was used for RNA extraction, which included the ovipositor, muscle tissue, the anus and the last part of the digestive tract [9]. We chose to leave the gland intact because in Hv pheromone is only found in extracts with intact glands (Groot and Schal, unpubl. res.).

### RNA extractions

TRIzol Reagent (Invitrogen) was used to isolate the RNA according to the manufacturer's protocol with several modifications. The RNA was precipitated overnight at -20°C and the dried pellet was dissolved in 90 μl RNA Storage Solution (Ambion). An additional DNAse (Turbo DNAse, Ambion) treatment was included prior to the second purification step to eliminate any contaminating DNA. The DNAse enzyme was removed and the RNA was further purified by using the RNeasy MinElute Clean up Kit (Qiagen) following the manufacturer's protocol and eluted in 20 μl of RNA Storage Solution (Ambion). RNA integrity and quantity was verified on an Agilent 2100 Bioanalyzer using the RNA Nano chips (Agilent Technologies, Palo Alto, CA). RNA quantity was determined on a Nanodrop ND-1000 spectrophotometer. RNA extractions were generated and four extracts were pooled to maximize the possible number of gene objects to be found and minimize unique contigs.

### Construction of the cDNA library

For both the *Heliothis virescens *mixed larval stages (different larval instars) and treatments (e.g. exposure to plant secondary metabolites, insecticides, immune insult) and the pheromone gland tissue material a full-length enriched, directionally cloned, normalized cDNA library was generated using a combination of the SMART cDNA library construction kit (Clontech) and the Trimmer Direct cDNA normalization kit (Evrogen) generally following the manufacturer's protocol but with several important modifications. In brief, 2 μg of total RNA was used for each cDNA library generated. Reverse transcription was performed with a mixture of several reverse transcription enzymes (ArrayScript, Ambion; BioScript, Bioline; PrimeScript, TaKaRa; SuperScript II, Invitrogen) for 1 h at 42°C and 90 minutes at 50°C. Each step of the normalization procedure was carefully monitored to avoid the generation of artifacts and overcycling. The optimal condition for ds-cDNA synthesis was empirically determined by subjecting the cDNA to a range of thermocycle numbers and their products checked by electrophoresis. The optimal cycle number (16 for the HvPGN sample) was defined as the maximum number of PCR cycles without any signs of overcycling.

To detect and classify both highly abundant transcripts and to generate a more complete transcriptome map of the gland, we generated and sequenced both a non-normalized and a normalized cDNA library of the pheromone glands of *Heliothis virescens*. An additional non-normalized cDNA library was generated for the *Heliothis virescens *pheromone gland tissue by primer extension with the MINT cDNA synthesis kit (Evrogen) according to the manufacturer's protocol. In order to compare the pheromone gland sequences to another tissue from the same species, we also generated a normalized cDNA library from larval tissue, essentially as described above for the *H. virescens *pheromone gland library.

Each of the resulting ds-cDNA pools for the different cDNA libraries was purified and concentrated using the DNA Clean and Concentrator kit (Zymogen) and size fractionated with SizeSep 400 spun columns (GE Healthcare) that resulted in a cutoff at ~200 bp. The full-length-enriched cDNAs were cut with *Sfi*I and ligated to pDNR-Lib plasmid (Clontech). Ligations were transformed into *E. coli *ELECTROMAX DH5α-E electro-competent cells (Invitrogen).

### Sequencing, Generation of EST Databases and Sequence Analysis

Plasmid minipreparation from bacterial colonies grown in 96 deep-well plates was performed using the 96 well robot plasmid isolation kit (NextTec) on a Tecan Evo Freedom 150 robotic platform (Tecan). Single-pass sequencing of the 5' termini of cDNA libraries was carried out on an ABI 3730 × l automatic DNA sequencer (PE Applied Biosystems). Vector clipping, quality trimming and sequence assembly using stringent conditions (e.g. high quality sequence trimming parameters, 95% sequence identity cutoff, 25 bp overlap) was done with the Lasergene software package (DNAStar Inc.).

In order to obtain a rough transcriptome coverage estimate for the pheromone gland library, we went through a series of search steps in order to i) obtain all hits against the conserved KEGG pathway database, and ii) estimate genome coverage by identifying the complete ribosomal protein dataset as compared to the full *Bombyx mori *set. Based on these findings we estimate the theoretical transcriptome coverage to be ~85% (70/79 *B. mori *ribosomal proteins were found).

We set up individual searchable databases for each of the species and used them to identify the genes we describe in more detail in the text. Blast searches were conducted on a local server using the National Center for Biotechnology Information (NCBI) blastall program. Homology and gene ontology (GO; http://www.geneontology.com), enzyme classification codes (EC) and metabolic pathway analysis of the assembled sequences were determined using the BLAST2GO software (http://www.blast2go.de). Sequences were searched against the NCBI non-redundant (nr) protein database using an E-value cut-off of 10^-3^, with predicted polypeptides of a minimum length of 18 amino acids.

Nucleotide sequences were analyzed using the commercial Lasergene Software package and the freeware BioEdit program. All EST sequences were submitted to Genbank (GenBank Acc: GR958232-GR972305). Genes were aligned by their amino acid sequences using the ClustalW function [97]. If necessary, alignments were then corrected by eye before being used for the contig and gene family analysis as well as the phylogenetic analyses.

To prepare annotated sequences for submission to GenBank, two strategies were used to remove redundant contigs in each library. First, protein translations of the contigs in each category were compared using the Multiple alignment program MAFFT http://align.bmr.kyushu-u.ac.jp/mafft/online/server/; this identified many contigs with predicted protein sequences that were >95% identical. Second, nucleotide consensus sequences of all 8310 contigs were subjected to two additional rounds of clustering using the program Sequencher (Gene Codes, Ann Arbor, Michigan). The first round grouped sequences with >85% identity over at least 30 nt using the "Clean data" option, and the second round used the same parameters with the "Dirty data" option. The new clusters were examined by eye and those with <95% identity over at least 30 nt were split again. 6318 of the original contig consensus sequences were not further grouped by this procedure; the remaining 1992 consensus sequences were grouped into 787 groups. All contigs combined by the MAFFT alignment were also combined by Sequencher. Assembled contigs were submitted to the TSA section of GenBank (Accession Numbers EZ407233-EZ407265). Additional sequences of individual clones for enzymes participating in the β-oxidation of fatty acids were deposited in GenBank (Accession Numbers GU205155 - GU205162).

### Phylogenetic reconstruction

The phylogenetic reconstruction implemented for the analysis of several proteins was performed using the Neighbour-Joining (NJ) method (TREECON). FAR and ACCase amino acid sequences were aligned by MAFFT http://align.bmr.kyushu-u.ac.jp/mafft/software/ and each visually inspected for regions of high quality alignment. The NJ consensus tree was generated with TREECON. Distance calculations were performed after Tajima & Nei and bootstrap analysis, running 1000 bootstrap samples [98]. Conserved residues in the alignments were highlighted with BOXSHADE 3.21 http://www.ch.embnet.org/software/BOX_form.html.

### *Bombyx mori *and *Agrotis segetum *pheromone gland ESTs

All of the available *Bombyx mori *pheromone gland library ESTs from Genbank (BP184340 - |BP182009; AV403746 - AV404455; EL928418 - EL930129; DC545768 - DC550742) and from the *Agrotis segetum *pheromone gland library ESTs (ES582293 - ES582156) were assembled with the same parameters as the *Heliothis virescens *ESTs to avoid any bias in subsequent data analysis.

### Best Bidirectional Hits and non-overlapping contigs

To identify best bidirectional hits between two sets of ESTs reciprocal tblastx WU-Blast analyses were performed. Custom Perl scripts then searched the blast output to find all cases where query "x" gave best hit "y" and query "y" gave best hit "x" (defined as Best Bidirectional Hit). For all of the inter-species comparisons (HvPG, BmPG, AsPG) the ValS value was 10. Perl scripts also identified HvPG ESTs that had no best bidirectional hit in HvLN (at ValS value = 500).

## Authors' contributions

HV carried out the molecular genetic studies, participated in the sequence alignment and in drafting the manuscript. AJH conducted the best bidirectional hits between the different databases. DGH participated in the sequence alignment, performed the statistical analysis and participated in drafting the manuscript. ATG conceived of the study, participated in its design and coordination and in the sequence alignment, and participated in drafting the manuscript. All authors read and approved the final manuscript.

## Supplementary Material

Additional file 1 **Top 250 highly expressed genes in Heliothis virescens pheromone glands**. Table containing the top 250 most highly expressed cDNAs identified in the Heliothis virescens pheromone gland library. cDNAs are ranked according to the total number of individual EST clones (No. of reads) representing the respective contig (SeqName), the best BLAST hit identified in the NCBI nr database (SeqDescr) and any associated Gene Ontology (GO) terms (GOTerms). Click here for file
